# Alcohol consumption and environmental influences on social relationships among Myanmar migrant workers in southern Thailand: A comprehensive study

**DOI:** 10.1016/j.jmh.2025.100376

**Published:** 2025-11-02

**Authors:** Kanit Hnuploy, Kittipong Sornlorm, Nirachon Chutipattana, Roshan Kumar Mahato, Rajitra Nawawonganun

**Affiliations:** a Faculty of science and technology, Suratthani Rajabhat University, Surat Thani, 84100, Thailand; b Faculty of Public Health, Khon Kaen University, Khon Kaen, 40002, Thailand; c Department of Community Public Health, School of Public Health, Walailak University, Nakhon Si Thammarat, 80161, Thailand; d Excellence Center on Public Health Research: EC for PHR, School of Public Health, Walailak University, Nakhon Si Thammarat, 80161, Thailand

**Keywords:** Alcohol consumption, Social relationships, Myanmar migrant workers, Migrant health, Workplace environment, Social integration

## Abstract

**Background:**

Myanmar migrant workers in Southern Thailand contribute significantly to the local economy but face challenges including harmful drinking patterns and poor environmental conditions that negatively impact their social interactions. Understanding these factors is crucial for developing effective interventions.

**Objectives:**

To examine how alcohol consumption and environmental conditions affect social relationship problems among Myanmar migrant workers in Southern Thailand.

**Methods:**

A cross-sectional survey was conducted among 516 Myanmar migrant workers who consume alcohol. Data were collected through structured interviews covering demographic characteristics, drinking patterns, and social relationship issues. Statistical analysis using Generalized Linear Mixed Models identified factors associated with social relationship problems.

**Results:**

Nearly two-thirds of participants (63.37 %, 95 %CI: 59.11–67.43) experienced at least one social relationship problem. Major risk factors included harmful drinking patterns (AOR=2.61, 95 %CI: 2.26–3.01), working in agriculture (AOR=2.04, 95 %CI: 1.17–3.55), and insufficient income (AOR=2.01, 95 %CI: 1.18–3.41). Workers living in rural communities (AOR=3.58, 95 %CI: 1.81–7.06) and those facing moderate-to-high workplace environmental problems (AOR=3.14, 95 %CI: 2.52–3.91) also showed significantly higher risks.

**Conclusion:**

This study highlights the critical roles of alcohol consumption and environmental factors in shaping social relationships among Myanmar migrant workers. Interventions should target harmful drinking behaviors, improve workplace conditions, and promote social integration to enhance the well-being of this vulnerable population.

## 1 Introduction

Hazardous alcohol consumption creates a major public health concern, ranking as the third primary cause of disease and disability globally. Alcohol accounts for around 4 % of global mortality, contributing to both acute and chronic health issues (World Health Organization, 2018). Additionally, alcohol-related diseases and injuries account for 4 to 5 percent of disability-adjusted life-years (DALYs), underscoring their significant impact on worldwide healthcare systems (Rehm et al., 2009). These adverse effects are not limited to individual health but extend to societal and economic factors (Crosland et al., 2024; MacKillop et al., 2022).

The negative impacts of alcohol usage are diverse, including physical health problems such as liver cirrhosis, cardiovascular disorders, and cancer, as well as several social problems (Im et al., 2023; World Health Organization, 2022). These include workplace inefficiency, spousal abuse, child maltreatment, accidents, and injuries. The fatal consequences of excessive alcohol consumption are underscored by the mortality associated with vehicular incidents, alcohol-fueled aggression, and self-inflicted damage. The financial consequences of excessive alcohol consumption are equally significant (Luangsinsiri et al., 2023). In the U.S. alone, these costs were estimated at $223.5 billion in 2006, equating to $746 per person, primarily driven by reduced workplace productivity, healthcare expenses, Law enforcement costs, and damages caused by alcohol-related incidents (Bouchery et al., 2011; Rehm et al., 2009).

Labor migrants often face individual issues associated with alcohol consumption. Alcohol consumption among migrants has been known as A resilience plan to address stresses like language hurdles, social isolation, prejudice, financial instability, and adverse working circumstances. In the initial phases of migration, alcohol consumption habits may be perceived as transient adaptive tactics (Escobar-Agreda et al., 2021). Nevertheless, when migrants adapt to host cultures where alcohol drinking is more socially common or prevalent than in their countries of origin, these transient habits may develop into habitual or harmful behaviors over time (Lee et al., 2007).

Societal and cultural factors strongly affect alcohol drinking behaviors within migrant communities. In the United States, Immigrant population constituted 13 % of the total population in 2010, with drinking trends among immigrants illustrating a complex interaction of their place of origin, socioeconomic level, cultural adaptation processes, and exposure to new social norms. (Bird et al., 2010; Grieco et al., 2012). The variety of experiences and backgrounds complicates the normalization of alcohol consumption patterns, as these are often influenced by factors such as religion, familial relationships, and access to community support systems.

Globally, labor migration continues to rise as people seek enhanced economic opportunities. According to the International Labor Organization (ILO), the number of global migrant workers increased from 150 million in 2009 to 164 million in 2017, illustrating the rising mobility of the workforce (International Labor Office, 2010; International Labor Organization, 2021). In Thailand, approximately 3.9 million migrant workers, mainly from Cambodia, Laos, Myanmar, and Vietnam, contribute significantly to the nation's agricultural, manufacturing, and fishing industries (United Nations Thematic Working Group on Migration in Thailand, 2019). While migration can bring economic benefits for both host and home countries, it also poses significant social and health challenges, including substance abuse, limited access to healthcare, and precarious legal statuses, all of which exacerbate the risk of migrant populations (Joppe, 2012).

In this study, the social context of Alcohol consumption indicates the close social surroundings where drinking takes place. The intake of alcohol in social contexts, such as parties with friends or cultural events, is commonly seen as a socially acceptable and pleasurable leisure activity. For problem drinkers, this habit may shift to solitary drinking, causing greater isolation and fractured relationships (Gonzalez and Skewes, 2012). Evidence shows that community characteristics and the proximity to alcohol outlets significantly affect drinking behaviors. Individuals living in areas with deficient infrastructure, including insufficient housing and subpar sanitation, are 150 % more likely to participate in heavy drinking than those in well-maintained communities (Bernstein et al., 2007).

The relationship between alcohol consumption, social relationships, and environmental factors among migrant populations is complex and multifaceted. Evidence shows that poor environmental conditions, including inadequate housing and workplace hazards, significantly influence drinking behaviors, with individuals in areas with deficient infrastructure being 150 % more likely to engage in heavy drinking (Bernstein et al., 2007). For migrant workers, these environmental stressors often combine with social isolation and occupational pressures to create a cycle where alcohol consumption further damages already strained relationships with family, colleagues, and employers (Moyce and Schenker, 2018; Sanchez, 2015). The social context of drinking also plays a critical role, as solitary drinking patterns associated with environmental stress can lead to greater isolation and fractured relationships (Gonzalez and Skewes, 2012). Moreover, factors such as income inequality and workplace stress have been linked to increased alcohol-related problems that subsequently affect social interactions (Grittner et al., 2013; Skogen et al., 2019). Understanding these interconnected pathways is crucial for developing comprehensive interventions that address not only individual drinking behaviors but also the environmental and social determinants affecting migrant workers' well-being.

Addressing alcohol abuse among migrants requires understanding the detailed connections between individual behaviors, environmental conditions, and social contexts. This study aims to examine the level of alcohol consumption and environmental factors that affect the social-relationship problem among Myanmar migrant workers in Southern Thailand, a population that is both integral to the local economy and vulnerable to significant social and health issue.

## 2 Research methodology/methods

**Design of the study:** This cross-sectional study aimed to explore the influence of alcohol consumption and environmental factors on social relationship problems among Myanmar migrant workers in Southern Thailand. Data were collected through structured interviews conducted from September 2023 to March 2024.

**Population and sample:** The study population included Myanmar migrant workers aged 18 years or older who had been employed in Southern Thailand for at least three months.

**Sampling frame and multi-stage procedure:** We employed a three-stage sampling strategy based on official registration data from the Ministry of Labor. According to April 2023 statistics, Southern Thailand contained Myanmar migrant workers across 14 provinces, providing a clear sampling frame for the first two stages.

Stage 1 - Province selection (Simple random sampling): Southern Thailand's 14 provinces were stratified into upper South (7 provinces) and lower South (7 provinces). Using simple random sampling by lottery method, we randomly selected one province from each stratum: Songkhla (lower South) and Surat Thani (upper South). These provinces were confirmed appropriate based on three criteria: (1) they contain the highest concentrations of registered Myanmar migrant workers (101,623 total: Songkhla 39,990 [39.35 %], Surat Thani 61,633 [60.65 %]), representing approximately 35 % of the region's migrant workforce, (2) they encompass diverse occupational sectors (agriculture, fishing, manufacturing) common throughout the region, and (3) they include both urban and rural settings, providing representative living and working conditions.
Stage 2 - Proportional allocation: Based on the calculated sample size of 516 participants (see below), we proportionally allocated participants by provincial worker population: Songkhla (203 participants, 39.35 %) and Surat Thani (313 participants, 60.65 %).
Stage 3 - Participant recruitment (Respondent-driven sampling): Within each province, we used respondent-driven sampling (RDS) to recruit participants. While provincial-level registration data provided our sampling frame for Stages 1–2, individual-level sampling frames do not exist because: (1) workers are geographically dispersed across numerous farms, factories, fishing ports, and residential areas, (2) many workers are mobile or in temporary housing not captured in static registries, and (3) language barriers and documentation concerns create access challenges. RDS is a validated probability-based method specifically designed for such hard-to-reach populations (Heckathorn, 1997). Initial "seed" participants (*n* = 10 per province) were identified through local health centers, NGOs serving migrant communities, and employers. Each seed received 3 referral coupons to recruit eligible peers. This continued in waves until reaching the target sample, with recruitment chains monitored to ensure diversity across occupational sectors and residential areas.

**Housing context:** In this study, "worker camps" refer to employer-provided collective housing facilities typically located on or near work sites, where workers share dormitory-style accommodations with communal facilities. These structured settings facilitate social interaction among workers, contrasting with independent housing arrangements (rental houses, apartments) where workers live with less organized community structure.

**Sample size calculation:** Sample size was determined using Hsieh's formula for multivariable analysis with binary outcomes (Hsieh et al., 1998). Using a prevalence of social relationship problems of 55 % based on prior WHO data (World Health Organization, 2005), significance level of 0.05, and power of 80 %, the required sample size was 516 participants.

**Outcome measurements:** The primary outcome was social relationship problems, assessed using four single-item questions adapted from the WHO Multi-Country Study framework (World Health Organization, 2005). Participants were asked to rate the quality of their relationships in four distinct domains: (1) relationships with neighbors, (2) relationships with colleagues, (3) relationships with family members, and (4) relationships with employers. Each item used a 3-point scale: good, normal, or poor.

For analysis, we created a binary outcome indicating whether participants experienced problems in at least one relationship domain. A participant was classified as having "social relationship problems" if they rated any of the four relationship domains as "poor." Those rating all domains as "good" or "normal" were classified as having "no social relationship problems." This dichotomization approach has been used in previous studies of migrant worker populations and aligns with public health approaches that identify individuals experiencing any social difficulties for targeted interventions.

Psychometric considerations: These four items represent distinct, directly-observed relationship domains rather than multiple indicators of a single latent construct. Therefore, exploratory factor analysis or internal consistency reliability (Cronbach's alpha) are not applicable to this measurement approach. Content validity was established through expert review by five specialists in migrant health and research methodology (Index of Item-Objective Congruence = 0.8–1.0). The questions demonstrated good face validity and comprehensibility in pilot testing with 30 Myanmar workers in Phuket province.

**Factors of interest and potential confounders:** Alcohol consumption patterns: Alcohol use was measured using the Alcohol Use Disorders Identification Test (AUDIT), a 10-item screening tool developed by the World Health Organization (Babor et al., 2001). The AUDIT assesses three domains: (1) alcohol consumption (items 1–3: frequency, typical quantity, and frequency of heavy drinking), (2) drinking behaviors and dependence symptoms (items 4–6: impaired control, increased salience, morning drinking), and (3) alcohol-related problems (items 7–10: guilt, blackouts, injuries, concern from others). Each item is scored 0–4, yielding total scores from 0 to 40. Scores were categorized as: low-risk drinking (0–7 points), hazardous drinking (8–15 points), harmful use (16–19 points), and alcohol dependence (≥20 points), following WHO guidelines (Babor et al., 2001). For multivariable analysis, we combined hazardous drinking and harmful use into a single category due to similar risk profiles.

Psychometric properties in our sample: The AUDIT demonstrated excellent internal consistency reliability in the main study sample (Cronbach's alpha = 0.97, *n* = 516) and good reliability in pilot testing (Cronbach's alpha = 0.84, *n* = 30 Myanmar workers in Phuket province). The questionnaire was administered in Burmese or Thai languages by trained bilingual interviewers who underwent standardized training. All 516 participants completed all AUDIT items with no missing data, indicating good comprehension and cultural appropriateness. AUDIT scores showed appropriate variability with no ceiling or floor effects, confirming the scale's discriminative ability in this population.

Environmental conditions: Housing and workplace environments were assessed using validated checklists adapted from occupational health research (Maeng et al., 2022; Palathoti et al., 2023). Housing conditions (8 items) evaluated ventilation, lighting, water supply, sanitation, overcrowding, noise, temperature control, and structural safety. Workplace conditions (9 items) evaluated ventilation, lighting, noise, temperature, sanitation facilities, overcrowding, chemical/dust exposure, physical hazards, and rest areas. Each item was rated 1–5 (none to very severe). Scores were categorized as low problems (housing: 8–18 points; workplace: 9–21 points) or moderate-to-high problems based on tertile distribution. Both scales showed excellent reliability in the main sample (housing: Cronbach's alpha = 0.92; workplace: Cronbach's alpha = 0.93) and acceptable reliability in pilot testing (housing: Cronbach's alpha = 0.76; workplace: Cronbach's alpha = 0.75).

Socioeconomic and demographic variables. Age was recorded in completed years and categorized into groups (18–25, 26–35, >35 years) for analysis. Gender was recorded as male or female. Marital status was classified as single, married/cohabiting, or divorced/separated/widowed. Education level was assessed as the highest level of formal schooling completed and categorized as primary or less, secondary, and higher education (high school, diploma, bachelor's degree or higher). Occupation type was classified based on primary employment sector: construction, agriculture/livestock, fishing, manufacturing, and services. Length of residence in Thailand was measured in years and categorized based on migration experience (<5 years, 5–10 years, >10 years). Monthly personal income and household expenditure were assessed as continuous variables and then categorized for analysis. Income categorization was based on prevailing wage structures across occupational sectors employing Myanmar migrant workers in Southern Thailand, with cutoff points reflecting economically meaningful distinctions observed in this population. Expenditure categorization was determined by examining proportional spending patterns to ensure adequate distribution of observations across groups while capturing variations in household economic burden. For categorical variables with sparse observations (*n* < 30 or <5 % of sample), categories were combined based on conceptual similarity to ensure statistical stability in multivariable models.

**Statistical analyses:** Descriptive statistics were used to summarize participant characteristics and the prevalence of social relationship problems. The dichotomous primary outcome (social relationship problems: yes vs. no) was analyzed using binary logistic regression. Bivariate analysis with logistic regression was conducted to explore associations between each independent variable and the outcome, and variables with a p-value below 0.25 were included in multivariable models (Hosmer et al., 2013). Generalized Linear Mixed Models (GLMM) were applied to account for the hierarchical nature of the data, including random effects for provinces. GLMM was chosen because our data exhibited clustering with individuals nested within 2 provinces (Songkhla: *n* = 203, 39.35 %; Surat Thani: *n* = 313, 60.65 %). Preliminary analysis revealed substantial between-province variation, as indicated by the intraclass correlation coefficient (ICC), demonstrating that a meaningful proportion of total variance in social relationship problems existed at the province level. This multilevel approach explicitly models within-cluster correlation, providing correct standard errors and avoiding Type I error inflation from ignoring clustering (Goldstein, 2011). Model diagnostics and validation: To verify the appropriateness of random effects modeling, we conducted a Hausman specification test comparing random effects with province fixed effects. The test results supported the random effects assumption, confirming that province-level effects were not significantly correlated with predictors. A likelihood ratio test validated the inclusion of random effects, demonstrating significant improvement over standard logistic regression and justifying the multilevel approach. Multicollinearity was tested using Variance Inflation Factor (VIF) with a threshold of <10. Independence of observations within clusters was verified after accounting for random effects. Model selection was guided by Akaike Information Criterion (AIC) and Bayesian Information Criterion (BIC), preferring models with the lowest values. Homoscedasticity of residuals and normality of random effects were checked visually using Q-Q plots. A complete case analysis was performed as there were no missing data reported. Adjusted odds ratios (AOR) with 95 % confidence intervals (CI) quantified associations, and statistical significance was set at p-value < 0.05. Data were analyzed using Stata 18.0 BE, licensed to Khon Kaen University.

## 3 Results

The study population consisted of 377 males (73.06 %) and 139 females (26.94 %). The mean age was 34.83 years (SD = 10.51), with a median age of 33 years. The age group distribution was as follows: 13 participants (2.52 %) were 19 years or younger, 154 participants (29.84 %) were aged 20 to 29 years, 210 participants (40.70 %) were aged 30 to 39 years, 124 participants (24.03 %) were aged 40 to 59 years, and 15 participants (2.91 %) were aged 60 years or older, with the age range spanning from 18 to 73 years.

The marital status distribution among the participants was as follows 168 individuals (32.56 %) were single, 182 individuals (35.27 %) were married with a marriage certificate, 123 individuals (23.84 %) were married without a marriage certificate, and 43 individuals (8.33 %) were divorced, widowed, or separated. Moreover, 252 individuals (48.84 %) reported not having children, while 264 individuals (51.16 %) reported having children. The distribution of the highest education level among the participants was as follows: 25 individuals (4.84 %) had no formal education, 152 individuals (29.46 %) completed primary school, 157 individuals (30.43 %) completed lower secondary school, 131 individuals (25.39 %) completed high school or its equivalent, 40 individuals (7.75 %) attained a diploma or its equivalent qualification, and 11 individuals (2.13 %) achieved a bachelor's degree or higher.

The main occupational groups among the participants were distributed as follows 183 individuals (36.17 %) were employed in agriculture and animal feeding sectors, 132 individuals (26.09 %) worked in manufacturing industry sectors, 105 individuals (20.75 %) were engaged in fishing sectors, 39 individuals (7.71 %) worked in households, 36 individuals (7.11 %) were employed in construction sectors, 7 individuals (1.38 %) worked in service industry sectors, and 4 individuals (0.79 %) were categorized under "others."

The average monthly income distribution among the participants was as follows: 11 individuals (2.13 %) reported having no income, 30 individuals (5.81 %) had an income <7500 Baht per month, 189 individuals (36.63 %) earned between 7500 and 9999 Baht per month, another 189 individuals (36.63 %) earned between 10,000 and 12,499 Baht per month, and 97 individuals (18.80 %) earned 12,500 Baht or more per month. The mean monthly income was 10,297.87 Baht (SD = 3136.42), with a median income of 10,000 Baht and a range from 0 to 20,000 Baht. The interpretation of the average monthly expenditure data indicates that the majority of participants, comprising 301 individuals (58.33 % of the sample), had monthly expenditures falling within the range of 5000 to 7499 Baht. This suggests that a significant portion of Myanmar migrant workers in Southern Thailand allocate a substantial portion of their income towards living expenses. Additionally, 90 participants (17.44 %) reported monthly expenditures between 2500 and 4999 Baht, while 85 participants (16.47 %) reported expenditures of 7500 Baht or more. A smaller percentage of participants, 29 individuals (5.62 %), had monthly expenditures below 2500 Baht, while 11 individuals (2.13 %) reported having no monthly expenditure at all. The mean monthly expenditure for the entire sample was calculated to be 5535.85 Baht, with a standard deviation of 2098.12 Baht. The median monthly expenditure, representing the middle value of the dataset, was reported as 6000 Baht, with reported expenditures ranging from 0 to 12,000 Baht. Regarding the appropriateness of income among the participants, the majority, comprising 432 individuals (83.72 % of the sample), considered their income to be appropriate. Conversely, a smaller proportion, comprising 84 individuals (16.28 % of the participants), deemed their income to be inappropriate. This suggests that a significant portion of Myanmar migrant workers in Southern Thailand perceive their income as adequate for meeting their financial needs, while a minority may feel that their income falls short of meeting their expenses or expectations.

The data on the duration of work in Thailand indicates that the participants have varied lengths of stay in the country. A considerable proportion, accounting for 195 individuals (37.79 % of the sample), had worked in Thailand for 2 to 5 years, followed by 138 individuals (26.74 %) who had been working for 6 to 10 years. Additionally, 82 individuals (15.89 %) had a tenure of <2 years, while 101 individuals (19.57 %) had worked in Thailand for over 10 years. The mean duration of work in Thailand for the entire sample was calculated to be 6.82 years, with a standard deviation of 6.33 years. The median duration, representing the middle value of the dataset, was reported as 5 years, with reported durations ranging from 0.08 to 36 years. This suggests a diverse range of experiences and lengths of stay among Myanmar migrant workers in Southern Thailand. The data on the participants' expectations regarding their future duration of work in Thailand indicates a varied outlook. The majority of participants, comprising 247 individuals (47.87 % of the sample), anticipated working in Thailand for 2 to 5 more years, followed by 162 individuals (31.40 %) who expected to continue working for 6 to 10 more years. Additionally, 61 individuals (11.82 %) expected to stay for <2 more years, while 46 individuals (8.91 %) anticipated working in Thailand for over 10 more years. The mean expected future duration of work in Thailand for the entire sample was calculated to be 7.24 years, with a standard deviation of 8.83 years. The median expected duration, representing the middle value of the dataset, was reported as 5 years, with expected durations ranging from 0.08 to 60 years. This data suggests a range of expectations among Myanmar migrant workers regarding their future tenure in Thailand, with a substantial portion anticipating a continued presence in the country for several years.

The data on the status of migrant workers reveals that the majority, comprising 466 individuals (90.31 % of the participants), were classified as illegal migrants, while a smaller proportion, accounting for 50 individuals (9.69 % of the sample), had legal migrant status. Regarding the participants' working hours per day, the majority, constituting 387 individuals (75 % of the sample), reported working 8 h or less per day, while 119 individuals (23.06 %) worked >8 h daily. The mean working hours per day for the entire sample were calculated to be 8.25 h, with a standard deviation of 1.58 h. The median working hours per day, representing the middle value of the dataset, was reported as 8 h, with reported working hours ranging from 0 to 15 h. This data suggests that while the majority of participants adhere to standard working hours, a notable proportion work longer hours, potentially indicating strenuous working conditions or multiple job commitments. The data on the participants' working time per week indicates that the majority, comprising 427 individuals (82.75 % of the sample), worked 6 days or fewer per week. A smaller proportion, accounting for 79 individuals (15.31 % of the participants), reported working every day of the week, while a very small percentage, 10 individuals (1.94 %), reported not having any job. The mean working days per week for the entire sample was calculated to be 5.90 days, with a standard deviation of 1.06 days. The median working days per week, representing the middle value of the dataset, was reported as 6 days, with reported working days ranging from 0 to 7 days. This data suggests that the majority of participants work on a full-time basis, with a standard workweek ranging from 5 to 6 days, although a notable minority reported working every day. The data on sleep problems indicates that a significant majority of participants, comprising 316 individuals (61.24 % of the sample), reported experiencing issues with sleeping.

Regarding the types of community, the participants were distributed across various settings. The largest proportion, accounting for 286 participants (55.43 %) of the sample, resided in semi-urban and semi-rural communities, followed by 105 participants (20.35 %) in Myanmar workers' communities, 87 participants (16.86 %) in urban communities, and 37 participants (7.17 %) in rural communities. Only 1 participant (0.19 %) resided in other types of communities. In terms of residence, the majority of participants, constituting 372 individuals (72.09 %) of the sample, lived in worker camps, while smaller percentages resided in detached rental houses (61 participants, 11.82 %), apartments (74 participants, 14.34 %), shared rental houses (5 participants, 0.97 %), or other types of residences (4 participants, 0.78 %). Regarding environmental problems, the majority of participants reported low levels of environmental issues both in their housing (456 participants, 88.37 %) and workplace (466 participants, 90.31 %). However, a small proportion reported moderate to high environmental problems in housing (60 participants, 11.63 %) and the workplace (50 participants, 9.69 %). This suggests that while most participants experience satisfactory environmental conditions, a minority face more significant challenges in this regard (Table I).

**Table I:** Demographic and socio-economic characteristics among Myanmar migrant workers in Southern Thailand (*n* = 516).

Demographic and socio-economic characteristics		Number	Percentage
**Gender**			
	Female	139	26.94
	Male	377	73.06
**Age (year)**			
	≤19	13	2.52
	20–29	154	29.84
	30–39	210	40.70
	40–59	124	24.03
	≥60	15	2.91
	Mean (Standard deviation)	34.83 (10.51)	
	Median (Min, Max)	33(18, 73)	
**Marital status**			
	Single	168	32.56
	Married (With marriage certificate)	182	35.27
	Married (Without marriage certificate	123	23.84
	Divorced / Widowed / Separated	43	8.33
**Having children**			
	No	252	48.84
	Yes	264	51.16
**Highest education**			
	No formal education	25	4.84
	Primary school	152	29.46
	Lower Secondary School	157	30.43
	High school or equivalence	131	25.39
	Diploma or equivalence	40	7.75
	Bachelor’s degree or higher	11	2.13
**Main occupational groups**			
	Agriculture and animal feeding sectors	183	36.17
	Manufacturing industry sectors	132	26.09
	Fishing sectors	105	20.75
	Household	39	7.71
	Construction sectors	36	7.11
	Service industry sectors	7	1.38
	Others	4	0.79
**An average monthly income (Baht /Month)**			
	None	11	2.13
	< 7500	30	5.81
	7500 – 9999	189	36.63
	10,000 – 12,499	189	36.63
	≥ 12,500	97	18.80
	Mean (Standard deviation)	10,297.87 (3136.42)	
	Median (Min, Max)	10,000 (0, 20,000)	
**An average monthly expenditure (Baht /Month)**			
	None	11	2.13
	< 2500	29	5.62
	2500 – 4999	90	17.44
	5000 – 7499	301	58.33
	≥ 7500	85	16.47
	Mean (Standard deviation)	5535.85 (2098.12)	
	Median (Min, Max)	6000 (0, 12,000)	
**Appropriate income**			
	Appropriate	432	83.72
	Inappropriate	84	16.28
**Worked in Thailand (year)**			
	< 2	82	15.89
	2–5	195	37.79
	6–10	138	26.74
	>10	101	19.57
	Mean (Standard deviation)	6.82 (6.33)	
	Median (Min, Max)	5 (0.08, 36)	
**Expect that you will work in Thailand (year)**			
	< 2	61	11.82
	2–5	247	47.87
	6–10	162	31.40
	>10	46	8.91
	Mean (Standard deviation)	7.24 (8.83)	
	Median (Min, Max)	5 (0.08, 60)	
**Status of migrant**			
	Illegal	466	90.31
	Legal	50	9.69
**Working time per day (hour)**			
	No have job	10	1.94
	≤ 8	387	75.00
	> 8	119	23.06
	Mean (Standard deviation)	8.25 (1.58)	
	Median (Min, Max)	8 (0, 15)	
**Working time per week (day)**			
	No have job	10	1.94
	≤6	427	82.75
	Everyday	79	15.31
	Mean (Standard deviation)	5.90 (1.06)	
	Median (Min, Max)	6 (0, 7)	
**Problems with sleeping**			
	No	200	38.76
	Yes	316	61.24
**Types of the community**			
	Myanmar workers community	105	20.35
	Urban community	87	16.86
	Rural community	37	7.17
	Semi-urban, semi-rural community	286	55.43
	Others	1	0.19
**Types of residence**			
	Worker camp	372	72.09
	Shared rental house	5	0.97
	Detached rental house	61	11.82
	Apartment	74	14.34
	Others	4	0.78
**Environmental problems in housing**			
	Low	456	88.37
	Moderate and High	60	11.63
**Environmental problems in workplace**			
	Low	466	90.31
	Moderate and High	50	9.69

Table II presents the prevalence of alcohol consumption levels among Myanmar migrant workers in Southern Thailand. Of the total sample size of 516 participants, 377 individuals (73.06 %) were classified as engaging in low-risk drinking, with a confidence interval (CI) ranging from 69.05 % to 76.72 %. A smaller proportion of participants, 30 individuals (5.81 %), fell into the category of hazardous drinking, with a 95 % CI of 4.09 % to 8.20 %. Additionally, 34 individuals (6.59 %) were categorized as experiencing harmful alcohol use, with a 95 % CI of 4.74 % to 9.08 %. Finally, 75 participants (14.54 %) were classified as exhibiting alcohol dependence, with a 95 % CI of 11.74 % to 17.85 %. These findings provide insight into the patterns of alcohol consumption among Myanmar Migrant Workers in Southern Thailand, highlighting varying levels of risk associated with alcohol use within this population.

**Table II:** Prevalence of alcohol consumption levels among Myanmar migrant workers in Southern Thailand (*n* = 516).

Level of alcohol consumption	Number	Percentage	95 % CI
Low-risk drinking (≤7 scores)	377	73.06	69.05 to 76.72
Hazardous drinking (8 −15 scores)	30	5.81	4.09 to 8.20
Harmful use (16 −19 scores)	34	6.59	4.74 to 9.08
Alcohol dependence (≥20 scores)	75	14.54	11.74 to 17.85

Table III presents the prevalence of social relationship problems among Myanmar migrant workers who consume alcohol in Southern Thailand. Out of the total sample size of 516 participants, 327 individuals (63.37 %) reported experiencing at least one social relationship problem, with a 95 % confidence interval (CI) ranging from 59.11 % to 67.43 %. Specifically, 304 participants (58.91 %) reported facing relationship problems with neighbors, with a 95 % CI of 54.60 % to 63.09 %. Furthermore, 286 individuals (55.42 %) reported experiencing relationship problems with colleagues, with a 95 % CI of 51.09 % to 59.67 %. Additionally, 218 participants (42.24 %) reported encountering relationship problems with family members, with a 95 % CI of 38.04 % to 46.56 %. Lastly, 258 individuals (50.00 %) reported experiencing relationship problems with employers, with a 95 % CI of 45.68 % to 54.31 %. These findings shed light on the prevalence of various social relationship problems experienced by Myanmar migrant workers who consume alcohol in Southern Thailand, highlighting the multifaceted nature of social challenges within this population.

**Table III:** Prevalences of social relationship problems among Myanmar migrant workers who consume alcohol in Southern Thailand (*n* = 516).

Social Relationship		Number	Percentage	95 % CI
**Social Relationship Problems (≥ 1 problem)**		**327**	**63.37**	**59.11 to 67.43**
	- Relationship problems with neighbors	304	58.91	54.60 to 63.09
	- Relationship problems with colleague	286	55.42	51.09 to 59.67
	- Relationship problems with family	218	42.24	38.04 to 46.56
	- Relationship problems with employer	258	50.00	45.68 to 54.31

Model selection and variable screening. All variables were initially screened through bivariate analysis to identify potential associations with the outcome. Variables with p-values < 0.25 in bivariate analysis, along with those identified as theoretically important from prior literature, were entered into the multivariable model. A backward elimination procedure was applied to identify the most parsimonious model, sequentially removing variables with the highest p-values until all remaining variables achieved statistical significance (*p* < 0.05) or were considered essential confounders. Model fit was assessed using AIC and BIC, with preference given to models with lower values. The final multivariable model demonstrated good fit and discriminative ability.

Table IV presents the key factors associated with social relationship problems, defined as a binary outcome (yes/no). Participants were classified as having "social relationship problems" if they reported poor relationship quality in at least one of four domains: relationships with neighbors, colleagues, family members, or employers. Those reporting good or normal relationships in all four domains were classified as having no social relationship problems. After adjusting for potential confounders using multivariable analysis, several factors emerged as significant predictors of social relationship problems.

**Table IV:** Multivariable analysis of factors associated with social relationship problems among Myanmar migrant workers in Southern Thailand (*n* = 516).

**Factors**		**Number**	**% of Social Relationship Problems**	**Crude OR**	**AOR**	**95% CI**	**P-value**
**Level of alcohol consumption**							0.007
	Low-risk drinking	377	59.95	1	1		
	Hazardous drinking and Harmful use	64	79.69	2.62	2.61	2.26 to 3.01	
	Alcohol dependence	75	66.67	1.33	1.76	1.16 to 2.66	
**Occupational groups**							0.012
	Others	411	60.83	1	1		
	Agriculture and animal feeding sectors	105	73.33	1.77	2.04	1.17 to 3.55	
**Appropriate income**							0.009
	Appropriate	432	61.11	1	1		
	Inappropriate	84	75.00	1.90	2.01	1.18 to 3.41	
**Types of the community**							
	Myanmar workers community	286	53.85	1	1		<0.001
	Urban community	105	72.38	2.24	2.18	1.35 to 3.53	
	Rural communities and others	125	77.60	2.96	3.58	1.81 to 7.06	
**Types of residence**							0.002
	Worker camp	144	52.78	1	1		
	Others	372	67.47	1.85	1.88	1.26 to 2.78	
**Environmental problems in workplace**							<0.001
	Low	466	62.66	1	1		
	Moderate and High	50	70.00	1.39	3.14	2.52 to 3.91	

**Note:** The dependent variable is social relationship problems (binary: yes = poor relationship quality in ≥1 of 4 domains; no = good/normal relationships in all domains). Workplace and housing environmental conditions are measured using separate scales assessing physical environment quality (ventilation, lighting, noise, sanitation, overcrowding, etc.) and are distinct from the relationship quality measures used to define the outcome. AOR = Adjusted Odds Ratio; CI = Confidence Interval. All models adjusted for province clustering using GLMM with random effects.

Environmental and occupational factors showed the strongest associations with social relationship problems. Workers facing moderate-to-high workplace environmental problems had more than three times the odds of experiencing relationship difficulties compared to those in better conditions (AOR=3.14, 95 %CI: 2.52–3.91). Similarly, living in rural communities was associated with over three-fold higher odds (AOR=3.58, 95 %CI: 1.81–7.06) compared to residing in Myanmar worker communities.

Alcohol consumption patterns significantly influenced social relationships. Participants with hazardous drinking or harmful alcohol use had 2.6 times higher odds of relationship problems (AOR=2.61, 95 %CI: 2.26–3.01) compared to low-risk drinkers, while those with alcohol dependence showed 1.8 times higher odds (AOR=1.76, 95 %CI: 1.16–2.66).

Socioeconomic factors also played important roles. Agricultural workers had twice the odds of experiencing relationship problems (AOR=2.04, 95 %CI: 1.17–3.55) compared to other occupational groups. Workers reporting inadequate income similarly showed doubled odds (AOR=2.01, 95 %CI: 1.18–3.41). Additionally, those living outside worker camps had nearly twice the odds of relationship difficulties (AOR=1.88, 95 %CI: 1.26–2.78).

## 4 Discussion

This study analyzed the factors associated with social relationship problems among Myanmar migrant workers who consume alcohol in Southern Thailand. The findings revealed that hazardous alcohol consumption, occupational roles, income inadequacy, community types, residential settings, and workplace environmental conditions were significant contributors to these Issues. Each factor is discussed in detail below, with an explanation of its relationship to the outcome and supporting evidence from the literature.

Alcohol consumption impacts social relationships through several pathways. Cognitive and emotional impairment from alcohol reduces judgment and impulse control, increasing conflicts and aggressive behaviors (Rehm et al., 2009). Among migrant workers facing high stress, alcohol amplifies negative emotions and triggers disproportionate reactions. Behavioral changes from regular drinking neglecting responsibilities, reduced work performance, and financial strain—directly erode relationship trust (Vanheusden et al., 2008). When drinking becomes solitary coping for isolation, it further distances individuals from social networks (Gonzalez and Skewes, 2012). Cultural displacement also plays a role, as migrants separated from traditional supports may adopt drinking patterns that strain relationships within both ethnic and host communities (Lee et al., 2007).

Level of alcohol consumption: Hazardous drinking impairs judgment and emotional regulation, amplifying conflicts among already-stressed migrant workers (Rehm et al., 2009; Vanheusden et al., 2008). Moreover, among migrant populations, alcohol is often used as a coping mechanism for stress, loneliness, and cultural displacement, further complicating relationships (Lee et al., 2007). This was consistent with the study by Vanheusden, K. et al., who found that alcohol consumption was associated with higher rates of internalizing problems in nondrinkers and negative social exchanges in excessive drinkers (Vanheusden et al., 2008). These findings highlight the importance of targeted interventions, such as alcohol education programs and community support networks, to reduce the social consequences of hazardous drinking in this group.

Occupational groups: Elevated risk among agricultural workers reflects physically demanding conditions and minimal job security, leading to stress-related alcohol use (Moyce and Schenker, 2018; Ramos et al., 2019). In addition, this is consistent with Ramos, A. et al., who found an association between alcohol consumption in agriculture and animal husbandry and social relationship problems, particularly among Latino farmworkers in Nebraska (Ramos et al., 2019). Additionally, the isolated nature of agricultural work often limits opportunities for social interaction and support, making workers more vulnerable to relationship challenges. This suggests that improving working conditions and providing mental health support for workers in these sectors could reduce their social vulnerabilities.

Income adequacy: Participants reporting inadequate income had higher odds of experiencing social relationship problems. Financial insecurity creates significant stress, affecting an individual’s ability to meet basic needs and maintain stable family dynamics. Economic hardship has been linked to increased alcohol consumption as individuals turn to drinking to escape or numb their stress (Aung et al., 2020). In addition, income inequality was associated with alcohol consumption and related problems, particularly among those with social and economic disadvantages, and inequality influences negative mood and stress-related behaviors, which may lead to problems in social relationships in some contexts (Godoy et al., 2006; Grittner et al., 2013). This can lead to neglect of family responsibilities, conflicts with spouses or children, and strained workplace relationships. Policies aimed at ensuring fair wages and income stability could play a crucial role in reducing both alcohol misuse and its social consequences.

Types of community: Living in rural or urban communities, as opposed to Myanmar workers' communities, was associated with a higher likelihood of social relationship problems. Rural areas often lack recreational facilities and social infrastructure, which can lead to boredom and increased alcohol consumption (Bernstein et al., 2007). In contrast, urban environments may expose individuals to peer pressure and cultural norms that normalize alcohol use, further compounding social challenges (Lee et al., 2007). In addition, domestic violence and community drinking, access to alcohol and advertising were factors that promoted alcohol consumption and social problems in both areas, especially rural communities were associated (Amanuel et al., 2018; Castaldelli-Maia, 2023; Pillai et al., 2013). with problems in social relationships, such as domestic violence and community drinking. Migrant communities, however, often offer shared cultural norms and a sense of solidarity, which can mitigate these risks. This highlights the importance of creating inclusive community environments that balance cultural preservation with opportunities for social integration.

Types of residence: Participants residing outside worker camps were more likely to report social relationship problems. Worker camps, while often criticized for their physical conditions, provide structured social environments that can foster a sense of community and reduce isolation. In contrast, living in independent residences may lead to social disconnection and fewer opportunities for peer support (Moyce and Schenker, 2018). In addition, the relationship between the types of residence of Myanmar migrant workers who consume alcohol, with migrant workers who consume alcohol for positive social interactions, may face different social relationship problems depending on the region and type of work (Racal et al., 2020). The absence of community engagement can exacerbate feelings of loneliness, increasing the likelihood of conflicts within families and workplaces. These findings suggest that housing policies for migrant workers should balance individual autonomy with opportunities for communal activities.

Environmental problems in the workplace: Moderate to high levels of workplace environmental problems, such as noise, overcrowding, and inadequate facilities, were significantly associated with social relationship problems. Poor working conditions not only affect physical health but also contribute to psychological stress, which can spill over into social relationships. Workers exposed to such environments often report higher rates of irritability, frustration, and interpersonal conflicts (Adhikary et al., 2017; Bernstein et al., 2007). In addition, workplace factors that cause stress and imbalances between effort and reward can increase the risk of alcohol-related problems, which may affect social relationships, and reducing the distance from work to alcohol outlets can increase the chances of alcohol problems (Raza et al., 2023; Skogen et al., 2019). Addressing workplace conditions through regulations and employer-led initiatives can help mitigate these issues and improve both worker well-being and social cohesion.

Our findings align with studies of migrant populations globally. The association between hazardous drinking and social problems is consistent with research on Latino farmworkers in the U.S. (Ramos et al., 2019; Sanchez, 2015). However, our study reveals higher effect sizes for environmental factors compared to studies in high-income countries, possibly reflecting weaker labor protections in middle-income settings. The strong rural effect may be specific to Southeast Asia, where rural migrant communities lack the ethnic social infrastructure found in urban areas. These findings suggest that while alcohol-environment-social relationships are universal, the relative importance of specific factors varies by context.

Perspective on the interconnectedness of factors: These findings underscore the complex interplay between individual behaviors and environmental contexts in shaping social relationship problems among migrant workers. Alcohol consumption often interacts with occupational stress, financial hardship, and poor living and working conditions, creating a cycle of vulnerability. To tackle these issues, a comprehensive approach is needed, involving policy changes, community programs, and improvements in workplace conditions. Offering support systems that are sensitive to cultural differences can ease the challenges faced by migrant workers and foster healthier social connections.

Given the strong workplace environmental effects, employers should prioritize: (1) environmental improvements (ventilation, noise reduction, sanitation), (2) workplace alcohol screening and brief interventions, and (3) peer support networks. For rural areas showing highest risks, establishing community centers with alcohol-free activities and mobile health services is critical. Policy reforms should strengthen workplace safety enforcement, ensure fair wages to reduce financial stress, expand legal migration pathways (90 % were illegal), and integrate migrants into health insurance for alcohol treatment access. Targeted interventions for hazardous drinkers should include screening with AUDIT and brief counseling. Importantly, effective interventions must be multi-level, addressing individual behaviors, workplace conditions, community environments, and policies simultaneously.

## 5 Strength and limitation of the study

This study provides a comprehensive analysis of the factors associated with social relationship problems among Myanmar migrant workers who consume alcohol in Southern Thailand. With a large and diverse sample and the use of Generalized Linear Mixed Models (GLMM) to account for hierarchical data structures, the findings are robust and offer valuable insights into the interplay of individual behaviors, socio-economic conditions, and environmental influences. However, the cross-sectional design limits the ability to establish causality, and some unmeasured factors, such as cultural influences, may have influenced the outcomes. The reliance on self-reported data also posed challenges in capturing nuanced details about alcohol consumption and social relationships. Despite these limitations, the use of validated tools and rigorous analytical methods strengthens the reliability of the results. Future research employing longitudinal designs and qualitative approaches could further clarify causal pathways and enrich our understanding of these issues.

## 6 Conclusions

The study provides a comprehensive examination of the socioeconomic, occupational, and health-related characteristics of Myanmar migrant workers in Southern Thailand. Findings reveal significant patterns in monthly expenditure, with a majority of workers allocating substantial portions of their income towards living expenses. While most workers perceive their income as appropriate, a notable minority deems it inadequate. Duration of work varies widely among participants, highlighting the diverse experiences of migrants in Thailand. Legal vulnerabilities are prevalent, with the majority of workers classified as illegal migrants. The study also sheds light on working conditions, sleep problems, and alcohol consumption patterns among workers, indicating potential health and social concerns. Factors such as alcohol consumption levels, occupational groups, income adequacy, and environmental conditions in the workplace significantly influence social relationship problems among workers. Overall, the study underscores the complex issues faced by Myanmar migrant workers in Southern Thailand and emphasizes the need for targeted interventions to improve their well-being and living conditions.

## 7 Recommendations

Recommendations based on the findings regarding social relationship problems among Myanmar migrant workers in Southern Thailand include implementing alcohol awareness programs to educate workers about the risks of hazardous drinking, ensuring fair wages and access to employment opportunities to address income inadequacy, facilitating community integration to reduce social isolation, advocating for improved working conditions, addressing housing challenges, implementing environmental health interventions in workplaces, and developing integrated support services tailored to migrant workers' needs. These measures aim to enhance social well-being and contribute to more inclusive and equitable communities.

## CRediT authorship contribution statement

**Kanit Hnuploy:** Writing – review & editing, Writing – original draft, Visualization, Supervision, Methodology, Formal analysis, Data curation, Conceptualization. **Kittipong Sornlorm:** Writing – review & editing, Writing – original draft, Visualization, Supervision, Methodology, Formal analysis, Data curation, Conceptualization. **Nirachon Chutipattana:** Writing – review & editing, Writing – original draft, Visualization, Methodology, Formal analysis, Data curation, Conceptualization. **Roshan Kumar Mahato:** Writing – review & editing, Writing – original draft, Visualization, Methodology, Conceptualization. **Rajitra Nawawonganun:** Writing – review & editing, Writing – original draft, Visualization, Methodology, Formal analysis, Conceptualization.

## Declaration of competing interest

The authors declare that they have no known competing financial interests or personal relationships that could have appeared to influence the work reported in this paper.
